# Ir(III) Complexes with AIE Characteristics for Biological Applications

**DOI:** 10.3390/bios12121104

**Published:** 2022-12-01

**Authors:** Yu Pei, Yan Sun, Meijia Huang, Zhijun Zhang, Dingyuan Yan, Jie Cui, Dongxia Zhu, Zebing Zeng, Dong Wang, Benzhong Tang

**Affiliations:** 1 Center for AIE Research, College of Materials Science and Engineering, Shenzhen University, Shenzhen 518060, China; 2 Key Laboratory of Nanobiosensing and Nanobioanalysis at Universities of Jilin Province, Department of Chemistry, Northeast Normal University, 5268 Renmin Street, Changchun 130024, China; 3 Shenzhen Research Institute of Hunan University, Shenzhen 518000, China; 4 School of Science and Engineering, Shenzhen Institute of Aggregate Science and Technology, The Chinese University of Hong Kong, Shenzhen 518172, China

**Keywords:** aggregation-induced emission (AIE), Ir(III) complexes, PDT, targeting, specific responsive

## Abstract

Both biological process detection and disease diagnosis on the basis of luminescence technology can provide comprehensive insights into the mechanisms of life and disease pathogenesis and also accurately guide therapeutics. As a family of prominent luminescent materials, Ir(III) complexes with aggregation-induced emission (AIE) tendency have been recently explored at a tremendous pace for biological applications, by virtue of their various distinct advantages, such as great stability in biological media, excellent fluorescence properties and distinctive photosensitizing features. Significant breakthroughs of AIE-active Ir(III) complexes have been achieved in the past few years and great progress has been witnessed in the construction of novel AIE-active Ir(III) complexes and their applications in organelle-specific targeting imaging, multiphoton imaging, biomarker-responsive bioimaging, as well as theranostics. This review systematically summarizes the basic concepts, seminal studies, recent trends and perspectives in this area.

## 1. Introduction

Iridium(III) complexes have attracted significant scientific interest in various fields, ranging from photoelectric devices, catalysis and chemical sensing to biological applications, benefitting from their prominent photophysical properties. Ir(III) has a higher spin-orbit coupling (SOC) constant (4430 cm^−^^1^) than other transition metals (Ru(II): 990 cm^−^^1^, Rh(III): 1425 cm^−^^1^, Os(II): 3531 cm^−^^1^, Pt(II): 4000 cm^−^^1^) and the strong SOC effect can accelerate the intersystem crossing (ISC) process [1,2]. Moreover, Ir(III) can easily form multiple coordination compounds. The structures of auxiliary ligands that coordinate with the metal center are rich and diverse, resulting in efficient metal to ligand charge transfer (MLCT), ligand-centered (LC) and ligand to ligand charge transfer (LLCT) [3,4,5], which causes strong phosphorescent emission and long excited-state lifetimes [6]. Meanwhile, Ir(III) complexes possess tunable photophysical properties, satisfactory photostability and large Stokes shifts. On the basis of those features, in particular, Ir(III) complexes are receiving great attention as promising candidates for optical bioimaging and phototherapy [7,8,9].

Photodynamic therapy (PDT) has accurately eradicated tumors and reduced side effects in healthy tissues by controlling the area of light irradiation. In general, the tumor apoptosis caused by PDT goes through photochemical reactions between excited triplet states of photosensitizers (PSs) and cellular substrates or oxygen to generate cytotoxic reactive oxygen species (ROS). Typically, the activated PSs may migrate to their triplet excited state via ISC. After that, the triple-activated PSs further react with oxygen to afford ROS via electron transfer (type I) or energy transfer (type II) processes [10]. Of particular interest is the heavy atom effect of Ir atoms, which plays an important role in efficiently enhancing the ability of ISC and effectively improving the generation efficiency of ROS [11,12,13]. However, the aggregation-caused quenching (ACQ) effect caused by the aggregation of Ir(III) complexes in an aqueous physiological environment leads to a reduction in luminescence emission and the generation capacity of ROS, which further limits their practical applications in the biomedical field [14,15].

Fortunately, aggregation-induced emission (AIE) was proposed in 2001 to solve the problem of unsatisfactory emission of luminescent materials in the aggregated state [16]. The main mechanism of the AIE phenomenon is the restricted intramolecular motion (RIM), thereby blocking the nonradiative energy decay pathway and boosting the radiative channel [17]. In recent years, AIE luminogens (AIEgens) have attracted extensive attention in the field of bioimaging, sensing and therapy applications, thanks to their distinct properties, including excellent photobleaching resistance and reliable output signal in an aggregated state [18,19,20]. In particular, AIEgens have unique aggregation-enhanced theranostics (AET) properties, referring to the advantages of enhancements in ROS generation, nonlinear optical effects under aggregation-induced effects [21,22]. On the other hand, many cases of Ir(III) complexes with AIE properties have been reported to date [23,24,25,26] and the following design strategies are generally recommended: (i) introducing active units with AIE properties into Ir(III) complexes and (ii) restricting intramolecular motion of flexible rotating units (such as imine bonds of Schiff bases) through coordination with the central metal iridium. Ir(III) complexes are potential phosphorescent molecules for the construction of AIE materials via strategic modification of ligands [27]. Therefore, constructing AIE phosphorescent materials based on Ir(III) complexes is one of the most promising candidates in the field of photo-triggered theranostics.

**Scheme 1:**
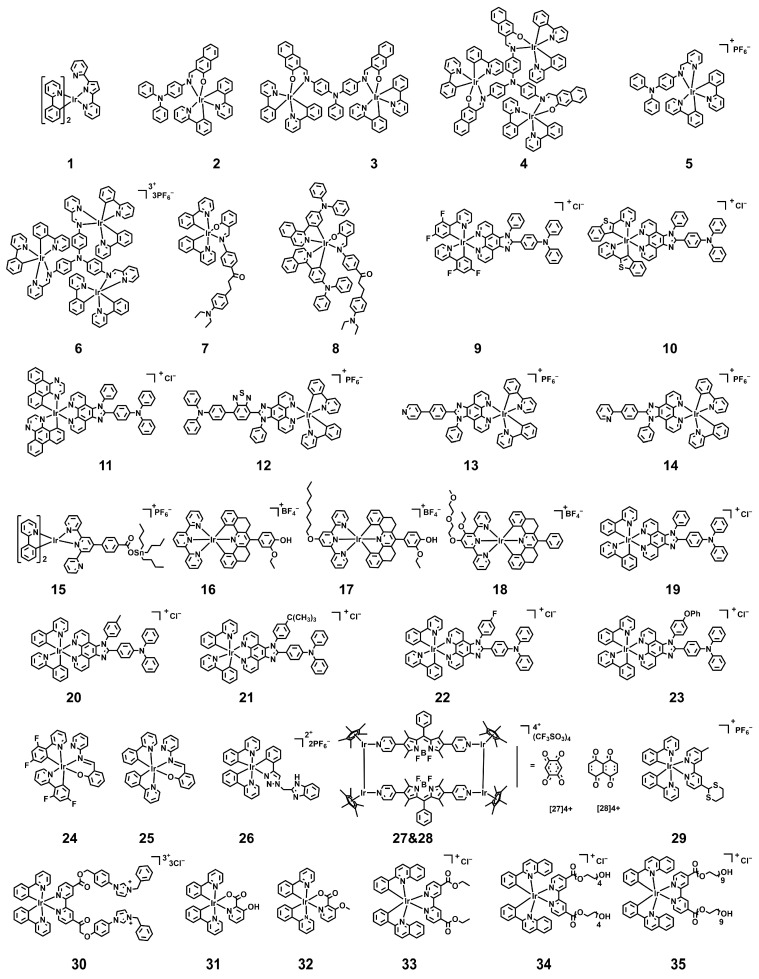
Chemical structures of Ir(III) complexes with AIE properties in biological field.

For bioimaging applications, Ir(III) complexes with the AIE property encounter aggregation spontaneously in a physiological environment, providing good signal-to-noise ratio and high sensitivity in specific responses to analytes. In addition, general Ir(III) complex PSs mainly exhibit two challenges in the field of PDT: (i) the original short excitation wavelength (300–450 nm) causes poor tissue penetration and adverse to deep tumor treatment [28,29]; (ii) ROS have a limited area of action region (20~40 nm) and short lifetime (<40 ns), so organelle-targeting is a desirable strategy to further enhance the efficacy of PDT [30,31,32]. At present, AIE-active Ir(III) complexes are still under development in the field of biological applications. Nonetheless, considerable progress has been made on AIE materials based on Ir(III) complexes to address the above two issues. For example, the excellent optical properties of Ir(III) complexes can overcome the problem of penetration depth of PSs through two-photon excitation [33,34,35,36]. Meanwhile, the positive charge and lipophilicity of the cationic Ir(III) complexes exhibit potential affinity to mitochondria [37,38,39,40]. This mini-review summarizes and presents the development of AIE-active Ir(III) complexes (Scheme 1) for bioimaging, sensing and therapy applications, in order to stimulate more researchers to enter this field.

## 2. Improving Photophysical Property of Ir(III) Complexes with AIE Activity

The main drawback of Ir-based PSs is that they are usually excited by UV/Vis light, which causes shallower penetration and limits the treatment of thicker and larger tumors [2,41]. Therefore, many concerns have focused on improving excitation and emission spectra of Ir(III) complexes [42].

### 2.1. Red-Shift Emission

**Figure 1:**
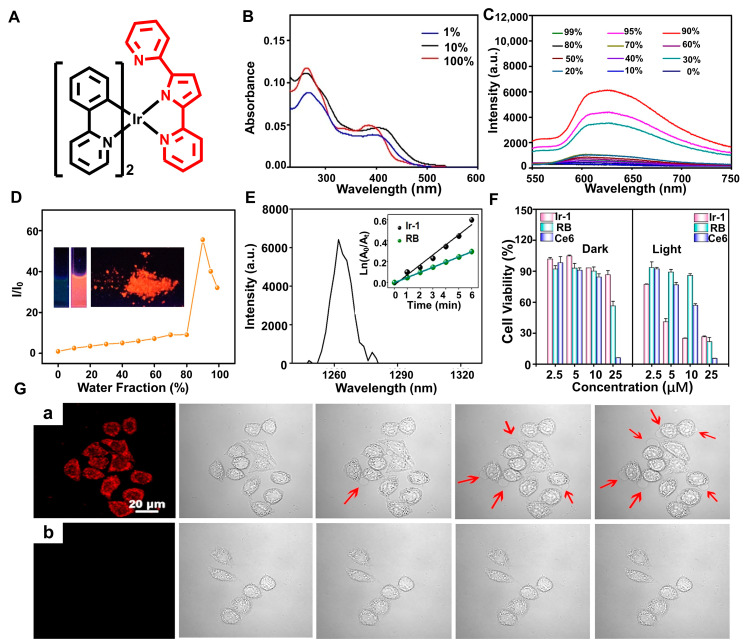
(**A**) The molecular structure of **1**. (**B**) Solution UV-vis absorbance spectra of **1 in** EtOH/water (fractions of EtOH 1%, 10% and 100%). (**C**) Phosphorescence spectra of **1** in EtOH/water (fractions of water ranged from 0 to 99%). (**D**) Variation in PL intensity in EtOH/water mixtures with *f*_w_. (**E**) The phosphorescence spectrum of ^1^O_2_. (**F**) The cytotoxicity and phototoxicity of **1**, Ce6 and RB to Hela cells. (**G**) CLSM images of Hela cells in the (a) presence (the red arrows: blebs were emerged around the cytomembrane) and (b) absence of **1** and their laser irradiation time-dependent bright-field images. Reprinted with permission from [44], copyright 2021, Wiley-VCH.

Making the emission red shift is a good way to reduce the interference of background antofluorescence and increase the depth of penetration [43]. Yang et al. [44] presented complex **1**, featuring AIE for image-guided PDT for cancer (Figure 1A). Complex **1** exhibited an absorption peak at ~384 nm (Figure 1B), with phosphorescence peaking at ~630 nm (Figure 1B). When the EtOH fraction was more than 20%, **1** exhibited weak phosphorescence intensity. Furthermore, the solid of **1** also demonstrated bright-red phosphorescence (in the inset of Figure 1D) and the solid-state phosphorescence quantum yield was 5.5%. Hence, **1** showed typical AIE features. It was observed that the ROS genergation efficiency of **1** was much better than that of Rose Bengal (RB), which is a commercially available and popular PS (Figure 1E). MTT assessment revealed that **1** displayed both high phototoxicity and low dark cytotoxicity (Figure 1F). Moreover, a cell-imaging experiment was conducted via the incubation of HeLa cells with **1**, as illustrated in Figure 1G. Cells can be clearly visualized with excellent image contrast to the cell background.

**Figure 2:**
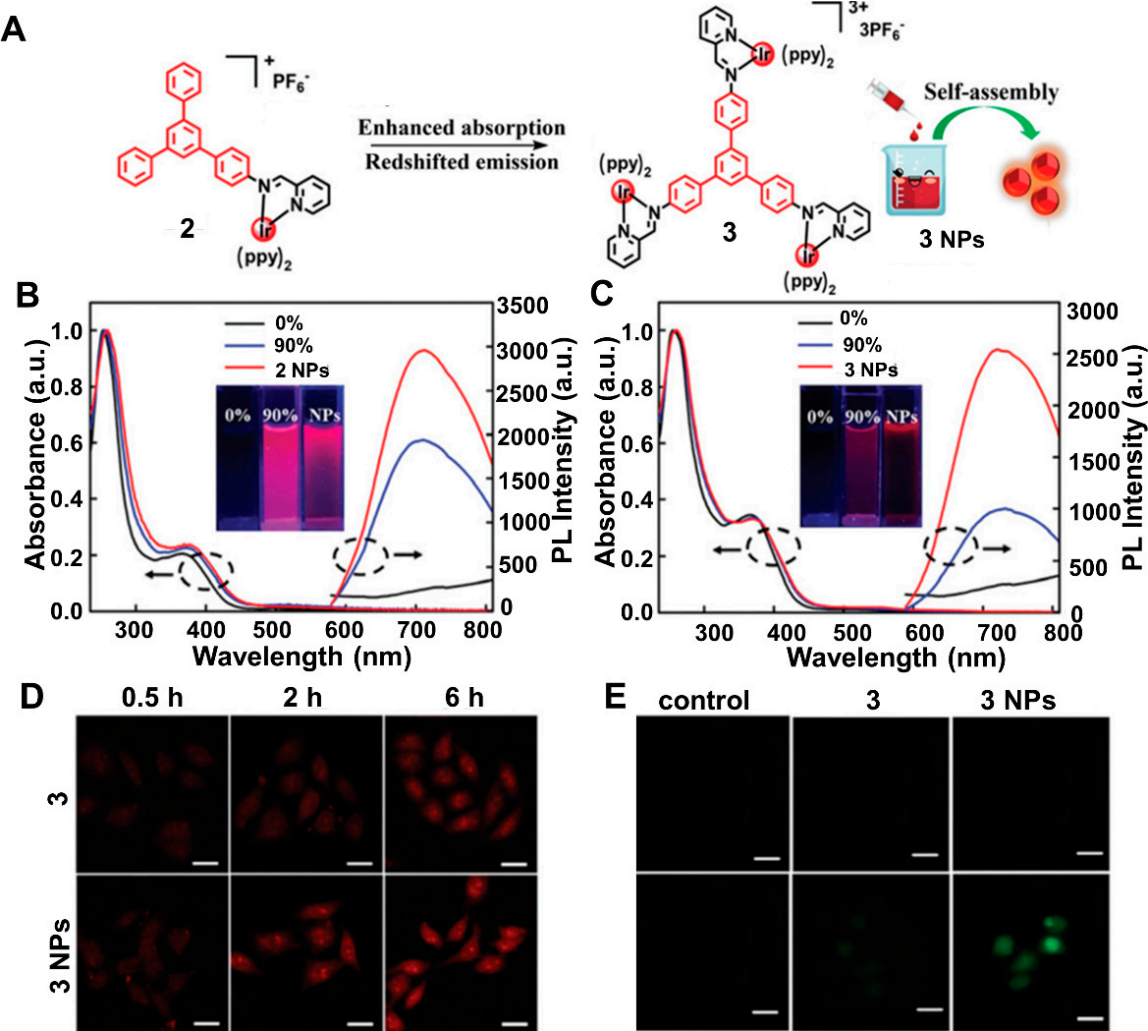
(**A**) Molecular structure of **2** and **3**. (**B**) UV–vis absorption spectra and emission spectra of **2**, **3** (DMSO, DMSO/water = 1/9) and corresponding NPs in water. (The arrows indicate the corresponding ordinate, The circles represent the absorption or emission curve of the materials). (**C**) CLSM images of Hela cells in the presence of **3** and **3 NPs**. (**D**) Cellular uptake of **3** and **3 NPs** detected by CLSM. (**E**) Confocal fluorescence images for the detection of ^1^O_2_ generation in HeLa cells treated with **3** and **3 NPs** under light. Reprinted with permission from [47], copyright 2020, Royal Society of Chemistry.

Compared with red emission, near-infrared (NIR) emission has better performance in overcoming autofluorescence interference, minimizing photodamage to cells and improving the signal-to-noise ratio (SNR) of imaging [45,46]. Li et al. designed and synthesized mono- and tri-nuclear NIR AIE cationic Ir(III) complexes and corresponding nanoparticles (NPs) **2** and **3** by self-assembling (Figure 2A) [47]. Further, **2** and **3** showed bright NIR emission at 710 nm and 730 nm when the water fraction reached 90%, showing typical AIE character (Figure 2B,C). The photoluminescence quantum yields (PLQYs) of **2** NPs and **3** NPs were 25% and 22% in water, which were higher than those of **2** and **3** (6% and 4%, respectively). Remarkably, the NPs possessed similar emission peaks while PL intensities were obviously enhanced. The greater biological NIR emission was valuable for efficient biological applications. Intracellular NIR emission was monitored, showing that **3 NPs** exhibited excellent imaging performance (Figure 2D). The ^1^O_2_ production was evaluated by using 2,7-dichlorodihydrofluorescein diacetate (DCFH-DA) as an indicator and bright-green fluorescence was observed with **3** and **3** NPs upon light irradiation (Figure 2E). These results proved that **3** NPs could be applied as an efficient PS for imaging-guided PDT.

### 2.2. Enhancing Molar Absorption Coefficient

**Figure 3:**
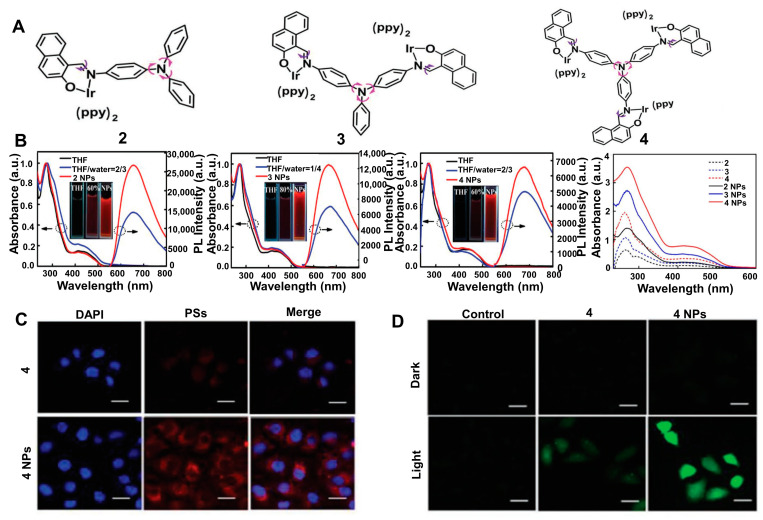
(**A**) Molecular structure of **4**, **5** and **6**. (**B**) UV–vis absorption spectra and emission spectra of **4**, **5**, **6** and corresponding NPs. (**C**) CLSM images of Hela cells in the presence of **6** and **6** NPs. (**D**) Confocal fluorescence images for the detection of ^1^O_2_ generation in Hela cells treated with **6** and **6** NPs. Reprinted with permission from [49], copyright 2019, Wiley-VCH.

In general, the upper limit of the output signal depends on the absorption capacity of the PSs. Actually, high brightness can be achieved by increasing the molar absorption coefficients. Therefore, improving absorptive capacity has been the focus of many efforts [48]. Zhang et al. proposed, for the first time, that additional metal centers further resulted in an increase in the molar absorption coefficient by increasing ^3^MLCT [49]. They designed the first example of an AIE-active multinuclear Ir(III) complex. Adding Ir(III) metal centers, the molar absorption coefficient increased (Figure 3A,B). Thus, **4** NPs, **5** NPs and **6** NPs showed higher PLQYs (33%, 15% and 35%, respectively) in water than those of **4**, **5** and **6** (11%, 5%, and 8%, respectively) in THF–water mixtures. Confocal laser scanning microscopy (CLSM) demonstrated the cellular uptake of PS and **6** NPs showed red fluorescence in cells (Figure 3C). The enhanced cellular uptake was highly advantageous to improve PDT. With light irradiation, green emission was observed from cells, indicating the presence of ^1^O_2_ generation (Figure 3D). This study offered a new method to improve the efficiency of PSs for clinical cancer treatments.

### 2.3. With the UCNPs

**Figure 4:**
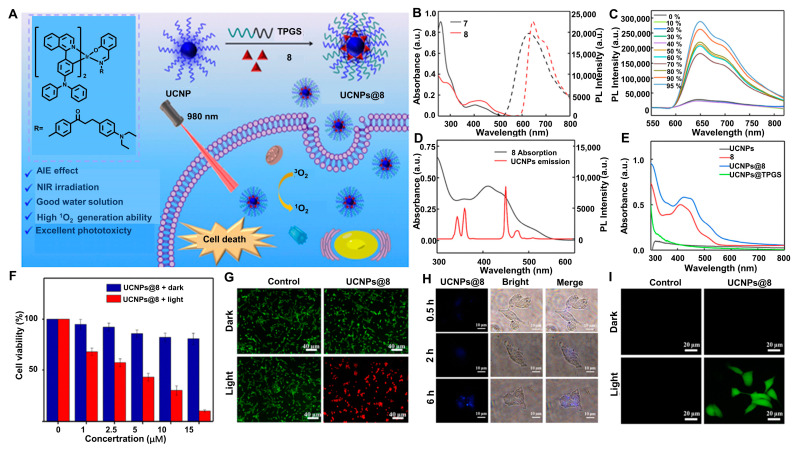
(**A**) Chemical structures of **UCNPs@8** and the application PDT. (**B**) Absorption and emission spectra of **7** and **8**. (**C**) PL spectra of **8** in CH_3_CN:H_2_O = 1:9 *v*/*v*. (**D**) The UV-vis absorption spectra of **8** (black line) and the PL spectra of UCNPs (red line). (**E**) The UV-vis absorption spectra of **8**, UCNPs, UCNPs@TPGS and **UCNPs@8**. (**F**) Cell viability of 4T1 cells treated with **UCNPs@8**. (**G**) Confocal fluorescence images of 4T1 cells co-stained with calcein-AM (live cells, green fluorescence) and propidium iodide (dead cells, red fluorescence) after treatment with **UCNPs@8**. (**H**) Cellular uptake of **UCNPs@8** detected by CLSM. (**I**) Confocal fluorescence images for detecting ^1^O_2_ generation in 4T1 cells treated with **UCNPs@8**. Reprinted with permission from [52], copyright 2022, Royal Society of Chemistry.

Upconversion nanoparticles (UCNPs) are desirable photoconversion materials for biosensing and biomedicine on account of their capability to transform the NIR photons to UV/visible photons [50,51]. Therefore, the conjugation of PSs to UCNPs presents an alternative to achieve NIR light-triggered generation of ROS. With this idea in mind, Liu et al. [52] employed AIE-active non-charged Ir(III) complexes integrated with UCNPs for the first time (Figure 4A). In UV-vis absorption spectra, **7** and **8** exhibited absorption peaks at ~425 nm. The PL spectra showed that **7** emitted at 640 nm and **8** emitted at 660 nm (Figure 4B), while the PLQYs were 16.6% and 18.9%, respectively. When the water fraction reached 50%, the emission intensity of **8** was greatly enhanced, which demonstrated that **8** had typical AIE features (Figure 4C). Further, **8** was then encapsulated with UCNPs within d-α-tocopherol polyethylene glycol 1000 succinate (TPGS) to construct **UCNPs@8** as PSs for the ensuing in vitro experiments. The UV-vis absorption spectra of **UCNPs@8** showed a significant absorption at about 425 nm, corresponding to that of **8** (Figure 4D). To ensure that the energy transferred between **8** and UCNPs can be realized and **UCNPs@8** could be irradiated by NIR light, the PL spectrum of UCNPs was measured under laser irradiation (980 nm). The absorption band of **8** was in the UV and blue regions, overlapping with the emission bands of UCNPs (Figure 4E). Encouraged by the ^1^O_2_ generation ability of NIR-irradiated **UCNPs@8**, their ability to kill cancer cells through PDT was investigated. MTT assays indicated that **UCNPs@8** had considerable cytotoxicity (Figure 4F). To further evaluate the therapeutic effect of **UCNPs@8**, Calcein AM and propidium iodide (PI) co-staining assay showed that a number of cells died under 980 nm irradiation (Figure 4G). The cells treated by **UCNPs@8** exhibited strong green fluorescence (Figure 4I). All the results illustrated the feasible strategy that UCNPs and AIE-active **8** were encapsulated together to achieve effective energy transfer and exerted their respective advantages to construct NIR-irradiated PSs, **UCNPs@8**, which generated toxic ROS and thereby killed the cancer cells. This approach effectively overcame the problems of Ir(III) complexes, which own short-wavelength absorption [52].

### 2.4. Two-Photon Absorption (TPA)

**Figure 5:**
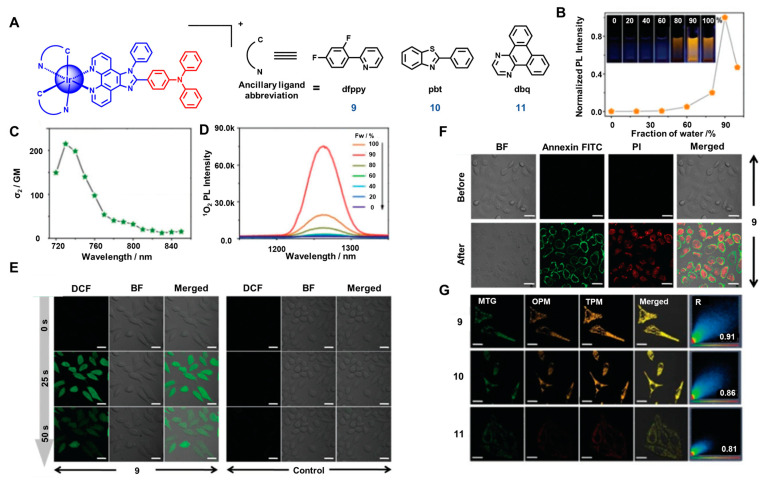
(**A**) Chemical structures of **9–11**. (**B**) PL spectra of **9** in DMSO/water mixed solvents with different water fraction. (**C**) TPA cross-sections of **9**. (**D**) ^1^O_2_ emission spectra in the presence of **9** and irradiation (405 nm) in varying fractions of water–DMSO mixture. (**E**) Confocal images of HeLa before and after TPA–PDT. (**F**) Annexin V-FITC/PI co-staining on HeLa cells before and after TPA–PDT. (**G**) Annexin V-FITC/PI co-staining on HeLa cells before and after TPA–PDT. Reprinted with permission from [60], copyright 2017, Royal Society of Chemistry.

In recent years, two-photon absorption (TPA) has emerged as an attractive protocol in bioimaging and PDT. Two-photon photodynamic therapy (TP-PDT) activates PSs by contemporaneously absorbing two photons [53,54,55,56]. It provides photodamage and phototoxicity as well as deeper penetration, weaker autofluorescence and lower photobleaching [57,58]. Developing Ir(III) complexes with TPA is a new strategy to enhance the efficiency of Ir-PSs. However, conventional PSs suffer from ACQ and reduced photocytotoxicity, resulting in inferior imaging and PDT efficacy. In summary, it is very prospective to construct Ir(III) complexes as AIE-active TPA PSs [59]. Liu et al. [60] conjugated triphenylamine with Ir(III) complexes to achieve the goals of AIE and TPA (Figure 5A). The PLQYs of **9** were 0.1% in DMSO. Further, **9** performed obvious AIE behavior and the TPA cross-sections (ð_2_) were better than the previous reported TPA organometallic molecular structure (Figure 5B,C). As anticipated, the ^1^O_2_ emission intensity enhanced as the water ratio of the mixture gradually increased from 0 to 90%, i.e., the strongest AIE effect (Figure 5D). Under two-photon laser irradiation, for the majority of cells treated with **9** (Figure 5E,F), the fluorescence intensity enhanced and significant annexin V-FITC (AV) and PI signals were detected, revealing that **9** possessed the highest potency of being an efficient TPA–PDT agent.

**Figure 6:**
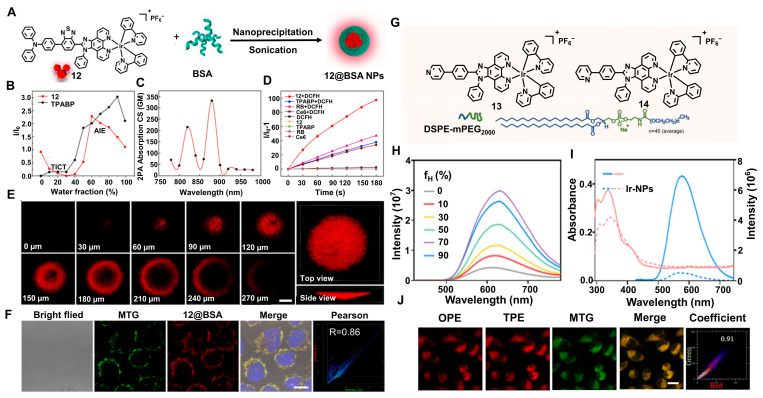
(**A**) Schematic illustration of the fabrication of **12@BSA NPs**. (**B**) Plots of the relative emission intensity (I/I_0_) versus water fraction. (**C**) Two-photon absorption cross-section of **12@BSA NPs**. (**D**) ROS detection in the presence of samples under laser illumination. (**E**) Two-photon images of 3D tumor cells incubated with **12@BSA NPs**. (**F**) Confocal images of MCF-7 cells after co-culture with **12@BSA NPs**. Reprinted with permission from [62], copyright 2022, Elsevier. (**G**) Chemical structures of **13** and **14**. (**H**) Phosphorescence spectra of **13** in H_2_O/DMSO mixtures. (**I**) The absorption and PL spectra of **13** and **13 NPs**. (**J**) The one-photon excitation (OPE)/two-photon excitation (TPE) confocal images of **13 NPs**. Reprinted with permission from [63], copyright 2021, Springer Nature.

However, TP-AIE-active Ir(III) complexes still have two main disadvantages: (i) Most activated TP-PSs undergo type II photochemical reactions to generate ROS. Their therapeutic effect is still limited by hypoxia. (ii) Poor water solubility makes it difficult to use in the clinic [61]. To address these issues, Jiang et al. designed the first type-I AIE PSs with the TP-PDT strategy, named **TPABP** and **12** (Figure 6A). To enhance biocompatibility and structural stability of **12**, bovine serum albumin (BSA) was added to formulate **12@BSA NPs**. **TPABP** and **12** exhibited the balance between the AIE feature and the typical TICT effect. When the water fraction was 0%, the PLQYs of **12** were about 9%. σ_2_ of **12@BSA NPs** was 340 GM at 880 nm (Figure 6B,C). The ROS generation of **12** was better than RB and Ce6 (Figure 6D). Under two-photon irradiation, a 3D tumor model was constructed to evaluate the penetrativity of **12@BSA NPs**. A penetration depth of 270 μm was measured by z-scanning (Figure 6E,F), which showed **12@BSA NPs** performed well [62]. Similarly, Cai et al. [63] synthesized two Ir(III) complexes (**13** and **14**) (Figure 6G) and **13** possessed the AIE feature (Figure 6H). Encapsulating **13** to DSPE-mPEG_2000_ to form nanoparticles (**13 NPs**) is a strategy to improve biocompatibility and the NP carrier did not influence the photophysical properties of **13** (Figure 6I). The cellular imaging capability of **13 NPs** was investigated using CLSM. Upon two-photon excitation, the red phosphorescent signal was stonger than that upon one-photon excitation (Figure 6J).

### 2.5. Multiphoton Absorption (MPA)

**Figure 7:**
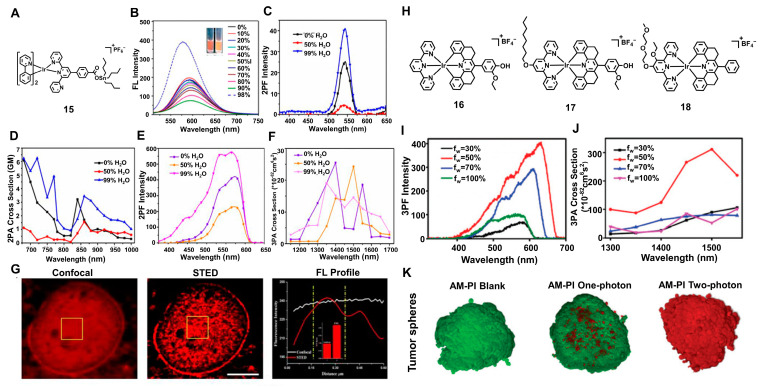
(**A**) The chemical structure of **15**. (**B**) PL spectra of **15** in different H_2_O fractions. (**C**) Two-photon fluorescence spectra of **15**. (**D**) Two-photon cross-section of **15**. (**E**) Three-photon fluorescence spectra of **15**. (**F**) Three-photon fluorescence spectra of **15**. (**G**) Two-photon fluorescence confocal imaging and stimulated emission depletion (STED) microscopy of **15** nuclear staining in A549 cells. Reprinted with permission from [64], copyright 2020, American Chemical Society. (**H**) The chemical structure of **16**, **17** and **18**. (**I**) Three-photon fluorescence spectra and (**J**) three-photon absorption cross-section of **16**. (**K**) 3D fluorescence images of 3D multicellular tumor spheroids under one-photon and two-photon laser irradiation. Reprinted with permission from [65], copyright 2022, Royal Society of Chemistry.

Compared with TPA, multiphoton absorption (MPA) improves the conversion efficiency through simultaneously absorbing several infrared photons. The two primary benefits of MPA have attracted attention: excitation wavelengths are in the near infrared (NIR) (700–900 nm) and the relationship between multiphoton absorption activity and excitation intensity is nonlinear [55]. Nevertheless, Ir(III) complexes activated by MPA are still very rare. Zhang et al. [64] combined the distinct merits of excellent photostability of infrared Ir(III) complexes, religious antibacterial ability of organotin(IV) compounds and MPA materials to design **15** and it showed obvious AIE behavior (Figure 7A,B). Using [Ru(byp)_3_]Cl_2_ as the standard, the PLQYs of **15** were 0.2 in water. The 2PA and 3PA cross-sections of **15** were 6.25 GM and 1.5 × 10^−81^ cm^6^ s^2^ per photon^2^ (Figure 7C–F). Under multiphoton confocal and STED microscopies, **15** showed superstability in nucleolus, which displayed that **15** had high signal-to-noise ratio (Figure 7G). Recently, Lu et al. [65] designed and synthesized a series of 3PA-Ir(III) complexes, named **16**, **17** and **18** (Figure 7H). Only **16,** which did not have a long alkoxy chain, showed AIE activity and excellent three-photon absorption properties (Figure 7I,J). Further, **16** also revealed the PLQYs, which were 1.4%. They displayed strong red emission under two-photon laser irradiation (820 nm), indicating the effectiveness of **16** in inducing cell death (Figure 7K).

## 3. Accurate Targeting of Ir(III) Complexes with AIE Activity

### 3.1. Targeting Mitochondria

It is well known that mitochondria are one of the most essential organelles. As the power workshop of cells, mitochondria provide most of the cellular ATP through pathways, such as pyruvate oxidation, fatty acid oxidation, tricarboxylic acid cycle (TCA cycle), and so on. On the other hand, mitochondria are the key to the apoptotic pathway. For instance, in extrinsic apoptosis, the mitochondrial outer membrane opens after signals, including intracellular Ca^2+^ overload and oxidation, which leads to the release of pro-apoptotic factors [66]. PSs act on mitochondria and make mitochondrial ROS burst, so mitochondria will be destroyed and then Cytochrome C, kinds of pro-apoptotic factors, will release, resulting in cancer cell death [67]. As a result, mitochondria-targeted PDT becomes an effective method to improve the treatment of cancer. The agents with delocalized positive charge are preferable to go through the mitochondrial lipid bilayer membrane due to the highly negative inner-membrane potential of mitochondria. Ir(III) complexes are demonstrated as mitochondrial targeting molecules applied in PDT, due to their lipophilic cationic properties.

Jiang et al. [62] proposed an Ir(III) complex with AIE characteristics, which was called **12**. Due to the feature that **12** was a lipophilic cation, there was an electrostatic interaction between **12 NPs** and mitochondria, holding a highly negative inner-membrane potential, which caused **12 NPs** to penetrate lipid bilayers and accumulate inside mitochondria (Figure 6I). Cai et al. [63] reported two Ir(III) complexes and both of them possessed AIE properties, good photosensitivity and mitochondria-targeting ability. Further, **13** was encapsulated with DSPE-mPEG_2000_ so that nanoparticles called **13 NPs** were formed. Taken up via caveolae-mediated endocytosis, **13 NPs** targeted mitochondria, which benefited from damaging mitochondria and enhanced antitumor effect (Figure 6M). Liu et al. [60] reported three different structural Ir(III) complexes **9–11**, which were PSs with AIE property and mitochondria-targeting capability, achieved though the endocytic pathway (Figure 5G). Interestingly, **9–11** exhibited more preferential photocytotoxicity towards HeLa cells than L02 cells.

**Figure 8:**
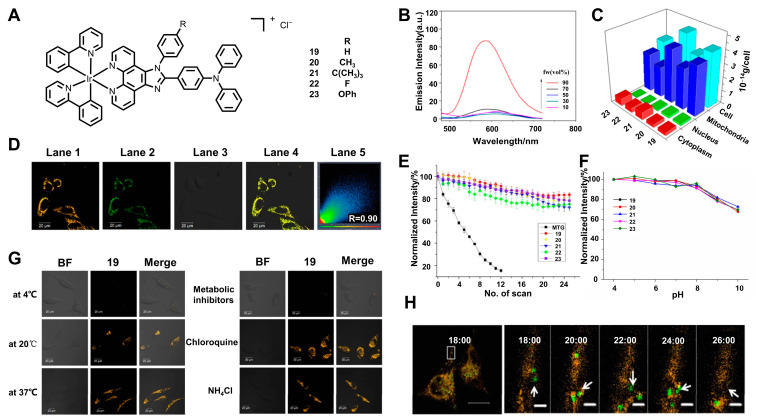
(**A**) Molecular structures of **19–23** complexes. (**B**) Emission spectra of **19** in DMSO-PBS mixtures with different water fractions. (**C**) Viability of HeLa cells incubated with 500 nM of **19–23**. (**D**) Confocal images of living HeLa cells incubated with **19** and their images. (**E**) Quantitative photobleaching results of **19–23** in HeLa cells. (**F**) Emission intensity of 10 μM of **19–23** at 590 nm under different pH. (**G**) Confocal luminescence image and bright-field images of living HeLa cells incubated with 500 nM **19** in DMSO-PBS under different conditions. (**H**) Confocal images of HeLa cells stained with **19** (500 nM, orange) and LTG (100 nM, green) in the presence of CCCP (10 μM). The regions (right) indicated in white boxes are enlarged from the shown area of this cell (left). The location that the white arrow pointed out indicated the occurrence of mitophagy. Reprinted with permission from [69], copyright 2016, Springer Nature.

In addition, the damaged mitochondria can be removed via mitophagy, a form of autophagy, which provides healthy surroundings in cells. During the autophagy process, a membrane structure forms the phagophore, which gradually expands and engulfs a portion of the cytosol or specific cargos and delivers them to the vacuole/lysosome for degradation [68]. The physiological changes are related to several diseases, so it is indispensable to monitor the mitophagy process. Jin et al. [69] synthesized five Ir(III) complexes, which possessed the AIE property (Figure 8A,B). These Ir(III) complexes, which were named **19–23**, were certified as specifically accumulated in mitochondria through mitochondrial co-localization imaging experiments with MitoTracker Green (MTG) and ICP-MS experiments (Figure 8C,D). The mechanisms of cellular uptake also proved that **19** used a non-endocytic energy-dependent active transport to cross the plasma membrane (Figure 8G). Thus, **19** successfully fulfilled the real-time monitoring of the mitophagy process, which was caused by CCCP (Figure 8H). In addition, **19–23** owned excellent stability during 10 scans, with a total irradiation time of ~150 s, as well as in a pH range from 4.0 to 8.0 (Figure 8E,F). Further, **19–23** were expected to be used as mitochondrion-specific probes in biological imaging and dynamic monitoring studies.

**Figure 9:**
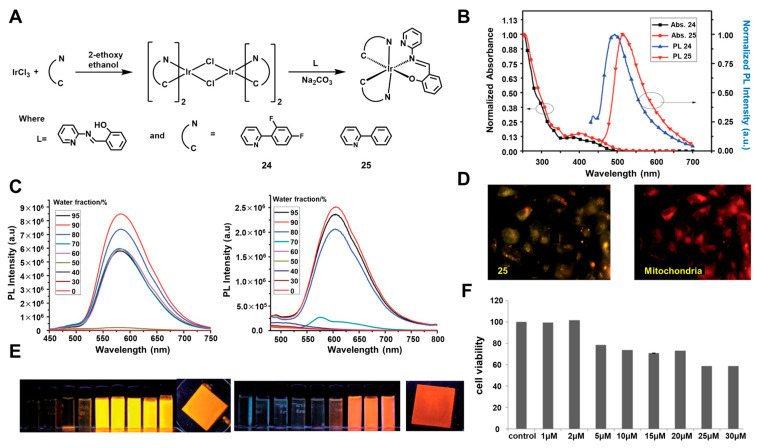
(**A**) Synthetic routes for complexes **24** and **25**. (**B**) Absorption and photoluminescence spectra of **24** and **25**. (The arrows indicate the corresponding ordinate, The circles represent the absorption or emission curve of the materials). (**C**) PL spectra of **24** and **25** in MeOH/water mixed solvents with different water fractions. (**D**) Intracellular compound localization of **25** in HOS cells. (**E**) Luminescent images of **24** and **25** in solutions with different water fractions and solid sample of **24** and **25** under irradiation with a 365 nm UV lamp. (**F**) Cytotoxicity assay for **25** in HOS cells. Reprinted with permission from [70], copyright 2017, Royal Society of Chemistry.

Ir complexes can be applied in mitochondrial staining simply. Alam et al. [70] proposed two AIE-active Ir(III) complexes (Figure 9A–C,E). The PLQYs for **25** were tested and found to be 6.03%. With poor ROS production capacity, **25** had lower cytotoxicity (Figure 9F) and **25** was useful for bioimaging. When **25** was incubated with HOS cells, the good fluorescence of **25** was only observed in mitochondria (Figure 9D). With AIE characteristics, low cytotoxicity and mitochondrion-targeted capability, **25** owned potential applications in bioimaging, particularly in mitochondrial staining.

### 3.2. Targeting Nucleolus

Ribonucleic acid (RNA) exists in biological cells and some viruses, as a viroid genetic information carrier. As the basis of life, RNA takes part in biological processes, such as gene transcription, post-transcriptional regulation of gene expression, protein synthesis based on gene information, etc. Thereinto, rRNA is responsible for protein synthesizing. rRNA gathers in the nucleolar region, which is a dark region under fluorescence microscopy. In recent years, the exploration of rRNA-selective probes for the nucleolar region becomes a meaningful topic. Holding more intense interaction with DNA than rRNA, small molecules are difficult to make use of as rRNA-selective probes. Ir(III) complexes have potential to construct a kind of rRNA-selective probe due to their greater interaction with intra-nuclear proteins [9].

**Figure 10:**
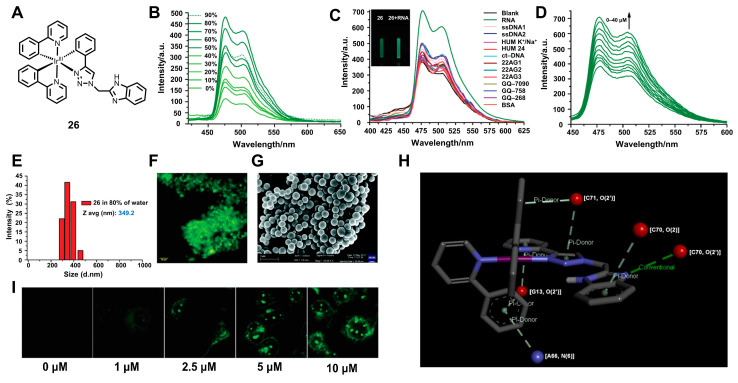
(**A**) Chemical structure of **26**. (**B**) THF–water mixtures with different water fractions (0 to 90%). (**C**) PL spectra of **26** (10 μM) upon addition of RNA, ctDNA, ssDNA, BSA and G-quadruplex DNA (4-fold) in CH_3_CN/PBS buffer. (**D**) PL titration of **26** with RNA (0 to 4-fold) in CH_3_CN/PBS buffer. (**E**) DLS profile of **26**. (**F**) Fluorescence and (**G**) SEM images of **26** (10 μM). (**H**) View of the interactions of **26** with nearby pyrimidine and purine bases of rRNA in docked structure. (**I**) Fluorescence microscopy images of HeLa cells stained with different concentrations of **26**. Reprinted with permission from [71], copyright 2018, Royal Society of Chemistry.

Based on the previous work, Sheet et al. [71] synthesized the **26** Ir(III) complex with benzimidazole-substituted 1,2,3-triazole-pyridine ligand (BiPT), which showed green luminescence (Figure 10A). The mechanisms of the AIE property of **26** are that **26** formed into aggregated particles in a CH_3_CN–water mixture and were verified by fluorescence, DLS and SEM images (Figure 10B,E–G). The PLQYs of **26** were found to be 0.016 in CH_3_CN at the same time. Noteworthily, a large portion of **26** was found to enter the cell nucleoli region after being incubated with Hela cells in co-staining experiments while faint green luminescence was still observed in the cytoplasm, which meant that **26** selectively targeted the nucleoli where most RNA was assembled (Figure 10I). Compared with ctDNA, ssDNA, G-quadruplex DNA and BSA, there was stronger interaction with **26** and RNA (Figure 10C). The results of Autodock Tools suggested that **26** combined with the purine and pyrimidine bases of RNA via supramolecular N–H∙∙∙π and π–π interactions (Figure 10H). Exhilaratingly, PL intensity of **26** elevated gradually with an enhancement in rRNA concentration (Figure 10D). Thereout, **26** was the first Ir(Ⅲ) complex reported as an rRNA-selective probe.

### 3.3. Selective Toxicity of Cells

**Figure 11:**
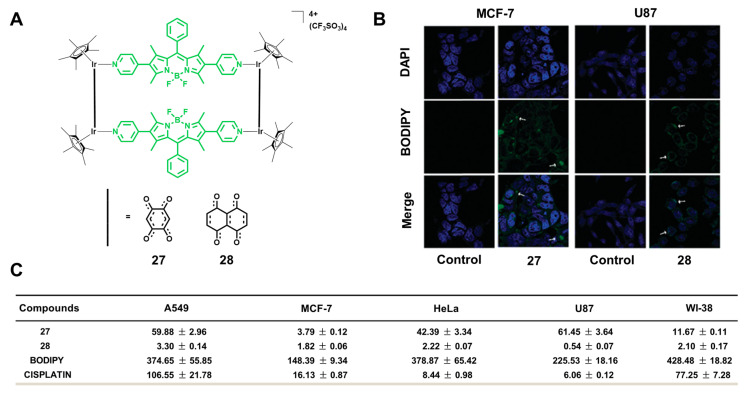
(**A**) Chemical structures of complexes **27** and **28**. (**B**) Left: MCF-7 cells treated with 1 mM **27**. Right: U87 cells treated with 0.75 mM **28**. (**C**) IC_50_ (μM) of **27–28**, BODIPY and cisplatin in different cell lines. Reprinted with permission from [72], copyright 2016, Royal Society of Chemistry.

Selective anticancer activity is one of the standards to estimate anticancer drugs. Damaging cancer cells and keeping normal cells well simultaneously are what researchers are trying to achieve. Ir(III) metalla-rectangles **27** and **28,** which consist of a BODIPY-based linker, were reported by Gupta et al. (Figure 11A). The white arrows showed **28** aggregated in the form of tiny granules, namely the AIE phenomenon (Figure 11B). Through initial biological screening, the selective toxicities of **27** against MCF-7 and **28** against U87 cells were proved (Figure 11B,C). The toxicity against cancer cell lines of **27** and **28** was stronger than that against normal cells. The complexes had different activities to different cell lines, attributed to various intracellular molecular targets, which demands further study [72].

## 4. Specific Response of Ir(III) Complexes with AIE Activity

In recent years, Ir(III) complexes with AIE properties have attracted more attention as photoluminescent probes in bioimaging, owing to their high photophysical performance (such as large Stokes shifts, tunable photophysical properties, long excited-states lifetime, satisfactory photostability, etc.). So far, several specific responsive probes based on Ir(III) complexes with AIE activity have been reported for bioimaging applications. The detailed structures and photophysical properties of these probes are summarized and discussed in the following sections.

### 4.1. Perchlorate (ClO_4_^−^)

**Figure 12:**
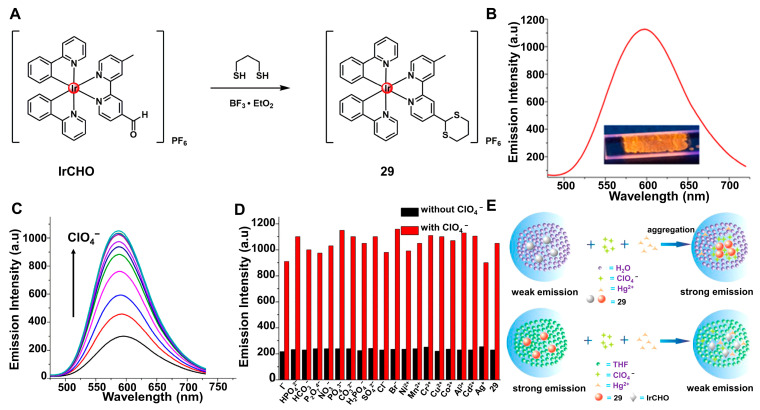
(**A**) Synthetic route of **29**. (**B**) The emission spectrum of **29** in solid state. Inset: emission image of **29** in solid state under 365 nm UV illumination. (**C**) Emission spectrum of **29** upon addition of NaClO_4_^−^ in HEPES buffer solution, λ_ex_ = 388 nm. (**D**) PL spectrum of **29** towards other ions in HEPES buffer solution at room temperature. (**E**) The mechanics of **29** in presence of Hg(ClO_4_)_2_ in THF and in H_2_O. Reprinted with permission from [75], copyright 2016, Elsevier.

Perchlorate (ClO_4_^−^) salts are widespread and relatively stable in foods and water samples [73]. ClO_4_^−^ competitively disrupts thyroid uptake of iodine and accumulates in the human body due to its similar ionic radius with iodide, resulting in adverse consequences, such as iodide deficiency and goiter, even brain impairment [74]. Hence, it is necessary to detect ClO_4_^−^ in water and monitor it in cells. An AIE-active complex **29** based on the Ir(III) complex for specific sensing of ClO_4_^−^ in cells reported by Chao et al. in 2016 was rationally designed by introducing the thioacetal group [75]. The phosphorescence quantum yield of **29** in solution was 1%, which increased to 2% in the solid state due to the aggregation of nanoparticles (Figure 12B). In particular, **29** exhibited a specific response to ClO_4_^−^ in HEPES buffer (Figure 12C,D) and the luminescence turn-on response was not interfered with by Hg^2+^, although the thioacetal group can undergo a thioacetal deprotection reaction [76]. Further, **29** and ClO_4_^−^ lead to enhanced-emissive nanoparticles in water, while the reaction of **29** and Hg^2+^ generated weak emission (Figure 12E). Moreover, the binding rate of **29** and ClO_4_^−^ was faster than the thioacetal deprotection reaction of Hg^2+^. The application of **29** in imaging ClO_4_^−^ in living cells was further investigated, where a strong red phosphorescent emission was observed in the cytoplasm.

**Figure 13:**
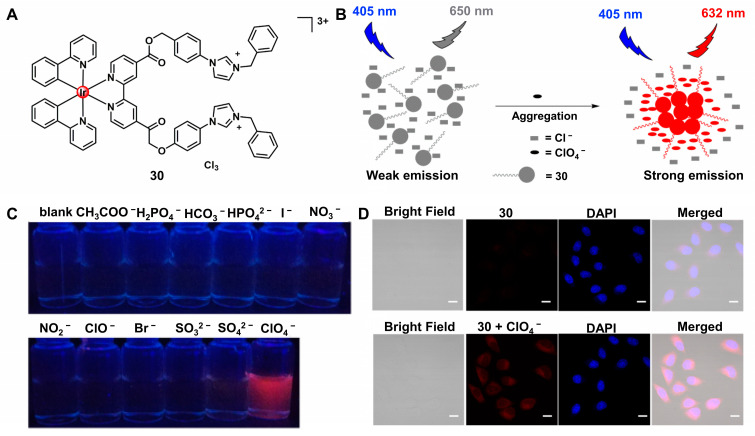
(**A**) The chemical structure of **30**. (**B**) Proposed possible processes of ClO_4_^−^ with **30** in H_2_O. (**C**) Corresponding images of ClO_4_^−^ and other anions under 365 nm UV illumination. (**D**) CLSM photographs of HeLa cells. Reprinted with permission from [77], copyright 2017, Elsevier.

In 2017, Chao et al. [77] first reported a water-soluble Ir(III) complex as a probe of ClO_4_^−^ based on the AIE phenomenon. Complex **30** had two imidazolium-like groups to improve positive charges and solubility (Figure 13A). A possible mechanism was presented that ClO_4_^−^ induced **30** and aggregated in water due to the exchange between Cl^−^ and ClO_4_**^−^**, resulting in an enhanced emission (Figure 13B). Furthermore, **30** was used for cell imaging and showed strong red emission in the presence of ClO_4_^−^ in HeLa cells (Figure 13D).

### 4.2. BSA

**Figure 14:**
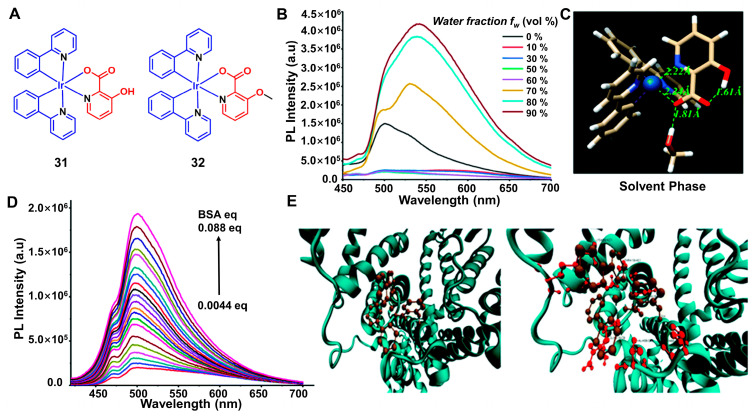
(**A**) The chemical structures of complexes **31** and **32**. (**B**) PL spectrum of **31** in MeOH with different water concentration. (**C**) DFT optimized structure of **31** in MeOH. (**D**) PL intensity of **31** (1 × 10^−5^ M) with gradually increasing BSA. (**E**) Alpha helices are binding partners of the ligand in the binding pocket and hydrogen bonding interactions of **31** with the residues of the proteins. Reprinted with permission from [80], copyright 2020, Royal Society of Chemistry.

BSA is a globular protein and has an important effect on biophysical functions, such as blood circulation and drug binding [78,79]. Two Ir(III) complexes, namely **31** and **32** (Figure 14A), were reported by Laskar et al. [80]. The emission intensity of **31** raised with the increasing concentration of water (Figure 14B). Compared with **32**, only **31** had AIE properties due to the restriction of intramolecular motion of the picolinate group (Figure 14C). Moreover, the interaction of **31** with BSA resulted in green emission (Figure 14D). The mechanism of **31** as a specific probe for sensing BSA proved that the auxiliary ligand may bind to the protein molecule through molecular docking studies (Figure 14E).

### 4.3. Drug-Resistant Bacteria

**Figure 15:**
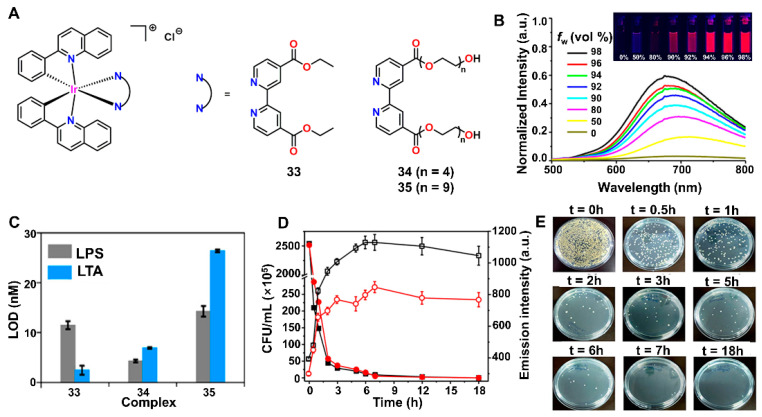
(**A**) The chemical structures of complexes **33–35**. (**B**) Emission spectrum of **34** in H_2_O–THF mixtures with different THF fractions. (**C**) Limit of detection (LOD) of LPS and LTA by the **33–35** confirmed from the linear fit curve of emission titration using fluorescence spectroscopy. (**D**) Kinetics of bacterial detection and growth inhibition. (**E**) Kinetics of growth inhibition for CRAB treated with **34** (15 μg/mL) in water. Reprinted with permission from [84], copyright 2020, American Chemical Society.

Antimicrobial resistance (AMR) has developed rapidly due to the overuse of medicines, leading to contaminating water bodies and posing a serious threat to public health [81,82]. Therefore, there is an urgent need for monitoring and eliminating drug-resistant bacteria to control waterborne diseases [83]. The work of Sasmal et al. is a good example of developing Ir(III) complexes with AIE properties for detecting and inhibiting drug-resistant bacteria [84]. Three cationic Ir(III) complexes (**33**–**35**) were synthesized as bacterial theranostic agents (Figure 15A). These complexes contained the same cyclometalated ligands and different auxiliary ligands. Further, **33** had two ethyl ester moieties in the auxiliary ligand, whereas **34** and **35** had bis-polyethylene glycol (PEG) to improve the biocompatibility and aqueous solubility. Both **33** and **34** were found to possess AIE active and highly selective for detecting LPS/LTA (Figure 15D). The PLQYs for **33**, **34** and **35** were determined to be 0.0072, 0.0091 and 0.0084, respectively. Moreover, the mechanisms of bacterial cells damaged by **33** and **34** suggest the disintegration of membrane integrity and generation of ROS (Figure 15E). This work is a platform that integrates the detection and elimination of bacteria into AIE-active Ir(III) complexes.

## 5. Conclusions

This review provides an overview of Ir(III) complexes with AIE properties for bioimaging, sensing and therapy applications in terms of extended absorption wavelength, precise targeting and specific response, respectively. By introducing various ligands into Ir(III) complexes, the photophysical properties could be improved to enhance the effect of bioimaging and therapeutics. These results are of great importance and promote the development of AIE materials based on Ir(III) complexes for biomedical applications.

However, there are still many limitations in the design strategy of efficient AIE-active Ir(III) complexes. Herein, we propose research directions where concerted efforts are required for AIEgens based on Ir(III) complexes in bioimaging and therapeutics. First, extending the absorption of Ir(III) complexes to NIR II will further improve the tissue-penetration depth and reduce side effects. Second, more attention should be placed on enhancing the targeting ability of AIE-active Ir(III) complexes and selectively releasing in the tumor microenvironment (TME) to improve the efficacy of tumor treatment. Third, the synergistic therapy of AIEgens could be further increased via exploiting the rational design of the Ir(III) complexes. We believe that AIE materials based on Ir(III) complexes would become a powerful tool for the biomedical field in the future.

## Data Availability

Not applicable.

## References

[1] Kober E.M., Meyer T.J. (1982). Concerning the absorption spectra of the ions M(bpy)_32_^+^ (M = Fe, Ru, Os; bpy = 2,2′-bipyridine). Inorg. Chem..

[2] You Y., Nam W. (2012). Photofunctional triplet excited states of cyclometalated Ir(III) complexes: Beyond electroluminescence. Chem. Soc. Rev..

[3] Flamigni L., Barbieri A., Sabatini C., Ventura B., Barigelletti F. (2007). Photochemistry and photophysics of coordination compounds: Iridium. Top. Curr. Chem..

[4] Zhao Q., Li F., Huang C. (2010). Phosphorescent chemosensors based on heavy-metal complexes. Chem. Soc. Rev..

[5] Alam P., Climent C., Alemany P., Laskar I.R. (2019). “Aggregation-induced emission” of transition metal compounds: Design, mechanistic insights, and applications. J. Photoch. Photobio. C Photoch. Rev..

[6] Ulbricht C., Beyer B., Friebe C., Winter A., Schubert U.S. (2009). Recent developments in the application of phosphorescent iridium(III) complex systems. Adv. Mater..

[7] Lee L.C.-C., Lo K.K.-W. (2022). Luminescent and photofunctional transition metal complexes: From molecular design to diagnostic and therapeutic applications. J. Am. Chem. Soc..

[8] Guan R., Xie L., Ji L., Chao H. (2020). Phosphorescent iridium(III) complexes for anticancer applications. Eur. J. Inorg. Chem..

[9] Caporale C., Massi M. (2018). Cyclometalated iridium(III) complexes for life science. Coordin. Chem. Rev..

[10] Gierlich P., Mata A.I., Donohoe C., Brito R.M.M., Senge M.O., Gomes-da-Silva L.C. (2020). Ligand-targeted delivery of photosensitizers for cancer treatment. Molecules.

[11] Huang H., Banerjee S., Qiu K., Zhang P., Blacque O., Malcomson T., Paterson M.J., Clarkson G.J., Staniforth M., Stavros V.G. (2019). Targeted photoredox catalysis in cancer cells. Nat. Chem..

[12] Shen J., Rees T.W., Ji L., Chao H. (2021). Recent advances in ruthenium(II) and iridium(III) complexes containing nanosystems for cancer treatment and bioimaging. Coordi. Chem. Rev..

[13] Liu Y., Song N., Chen L., Xie Z.-G. (2018). BODIPY@Ir(III) complexes assembling organic nanoparticles for enhanced photodynamic therapy. Chinese J. Polym. Sci..

[14] Mei J., Hong Y., Lam J.W.Y., Qin A., Tang Y., Tang B.Z. (2014). Aggregation-induced emission: The whole is more brilliant than the parts. Adv. Mater..

[15] Yuan Y., Xu S., Cheng X., Cai X., Liu B. (2016). Bioorthogonal turn-on probe based on aggregation-induced emission characteristics for cancer cell imaging and ablation. Angew. Chem. Inter. Ed..

[16] Luo J., Xie Z., Lam J.W., Cheng L., Chen H., Qiu C., Kwok H.S., Zhan X., Liu Y., Zhu D. (2001). Aggregation-induced emission of 1-methyl-1,2,3,4,5-pentaphenylsilole. Chem. Commun..

[17] Chen J., Law C.C.W., Lam J.W.Y., Dong Y., Lo S.M.F., Williams I.D., Zhu D., Tang B.Z. (2003). Synthesis, light emission, nanoaggregation, and restricted intramolecular rotation of 1,1-substituted 2,3,4,5-tetraphenylsiloles. Chem. Mater..

[18] Chen C., Ni X., Jia S., Liang Y., Wu X., Kong D., Ding D. (2019). Cancer immunotherapy: Massively evoking immunogenic cell death by focused mitochondrial oxidative stress using an AIE luminogen with a twisted molecular structure. Adv. Mater..

[19] Wang D., Tang B.Z. (2019). Aggregation-induced emission luminogens for activity-based sensing. Acc. Chem. Res..

[20] Kwok R.T.K., Leung C.W.T., Lam J.W.Y., Tang B.Z. (2015). Biosensing by luminogens with aggregation-induced emission characteristics. Chem. Soc. Rev..

[21] Zhang Z., Kang M., Tan H., Song N., Li M., Xiao P., Yan D., Zhang L., Wang D., Tang B.Z. (2022). The fast-growing field of photo-driven theranostics based on aggregation-induced emission. Chem. Soc. Rev..

[22] Song N., Zhang Z., Liu P., Yang Y.W., Wang L., Wang D., Tang B.Z. (2020). Nanomaterials with supramolecular assembly based on AIE luminogens for theranostic applications. Adv. Mater..

[23] Zhao Q., Li L., Li F., Yu M., Liu Z., Yi T., Huang C. (2008). Aggregation-induced phosphorescent emission (AIPE) of iridium(III) complexes. Chem. Commun..

[24] Pei Y., Xie J., Cui D., Liu S., Li G., Zhu D., Su Z. (2020). A mechanochromic cyclemetalated cationic Ir(III) complex with AIE activity by strategic modification of ligands. Dalton Trans..

[25] Li D., Li G., Xie J., Zhu D., Su Z., Bryce M.R. (2019). Strategic modification of ligands for remarkable piezochromic luminescence (PCL) based on a neutral Ir(III) phosphor. J. Mater. Chem. C.

[26] Di L., Xing Y., Yang Z., Qiao C., Xia Z. (2023). Photostable aggregation-induced emission of iridium(III) complex realizing robust and high-resolution imaging of latent fingerprints. Sens. Actuat. B Chem..

[27] Li G., Ren X., Shan G., Che W., Zhu D., Yan L., Su Z., Bryce M.R. (2015). New AIE-active dinuclear Ir(III) complexes with reversible piezochromic phosphorescence behaviour. Chem. Commun..

[28] He Z., Zhao L., Zhang Q., Chang M., Li C., Zhang H., Lu Y., Chen Y. (2020). An acceptor–donor–acceptor structured small molecule for effective NIR triggered dual phototherapy of cancer. Adv. Funct. Mater..

[29] Chelushkin P.S., Shakirova J.R., Kritchenkov I.S., Baigildin V.A., Tunik S.P. (2022). Phosphorescent NIR emitters for biomedicine: Applications, advances and challenges. Dalton Trans..

[30] Zhang P., Wu G., Zhao C., Zhou L., Wang X., Wei S. (2020). Magnetic stomatocyte-like nanomotor as photosensitizer carrier for photodynamic therapy based cancer treatment. Colloid. Surface. B Biointerfaces.

[31] Li B., Zhou Q., Wang H., Zha Y., Zheng P., Yang T., Ma D., Qiu L., Xu X., Hu Y. (2021). Mitochondria-targeted magnetic gold nanoheterostructure for multi-modal imaging guided photothermal and photodynamic therapy of triple-negative breast cancer. Chem. Eng. J..

[32] Smith R.A.J., Hartley R.C., Murphy M.P. (2011). Mitochondria-targeted small molecule therapeutics and probes. Antioxid. Redox Signal..

[33] McKenzie L.K., Sazanovich I.V., Baggaley E., Bonneau M., Guerchais V., Williams J.A.G., Weinstein J.A., Bryant H.E. (2017). Metal complexes for two-photon photodynamic therapy: A cyclometallated iridium complex induces two-photon photosensitization of cancer cells under near-IR light. Chem. Eur. J..

[34] Tian X., Zhu Y., Zhang M., Luo L., Wu J., Zhou H., Guan L., Battaglia G., Tian Y. (2017). Localization matters: A nuclear targeting two-photon absorption iridium complex in photodynamic therapy. Chem. Commun..

[35] Cho S., You Y., Nam W. (2014). Lysosome-specific one-photon fluorescence staining and two-photon singlet oxygen generation by molecular dyad. RSC Adv..

[36] Kuang S., Sun L., Zhang X., Liao X., Rees T.W., Zeng L., Chen Y., Zhang X., Ji L., Chao H. (2020). A mitochondrion-localized two-photon photosensitizer generating carbon radicals against hypoxic tumors. Angew. Chem. Inter. Ed..

[37] Cao R., Jia J., Ma X., Zhou M., Fei H. (2013). Membrane localized iridium(III) complex induces endoplasmic reticulum stress and mitochondria-mediated apoptosis in human cancer cells. J. Med. Chem..

[38] Zielonka J., Joseph J., Sikora A., Hardy M., Ouari O., Vasquez-Vivar J., Cheng G., Lopez M., Kalyanaraman B. (2017). Mitochondria-targeted triphenylphosphonium-based compounds: Syntheses, mechanisms of action, and therapeutic and diagnostic applications. Chem. Rev..

[39] Murase T., Yoshihara T., Tobita S. (2012). Mitochondria-specific oxygen probe based on iridium complexes bearing triphenylphosphonium cation. Chem. Lett..

[40] Chen Y., Rees T.W., Ji L., Chao H. (2018). Mitochondrial dynamics tracking with iridium(III) complexes. Curr. Opin. Chem. Biol..

[41] Li G., Zhu D., Wang X., Su Z., Bryce M.R. (2020). Dinuclear metal complexes: Multifunctional properties and applications. Chem. Soc. Rev..

[42] Wang J., Lu Y., McGoldrick N., Zhang C., Yang W., Zhao J., Draper S.M. (2016). Dual phosphorescent dinuclear transition metal complexes, and their application as triplet photosensitizers for TTA upconversion and photodynamic therapy. J. Mater. Chem. C.

[43] Zhang J., Zheng M., Zhang F., Xu B., Tian W., Xie Z. (2016). Supramolecular hybrids of AIEgen with carbon dots for noninvasive long-term bioimaging. Chem. Mater..

[44] Yang K., Zhou Y., Wang Y., Zhao S., Wu X., Peng X., Huang L., Jiang L., Lan M., Yi X.-Y. (2021). An iridium complex as an AIE-active photosensitizer for image-guided photodynamic therapy. Chem. Asian J..

[45] Dai J., Li Y., Long Z., Jiang R., Zhuang Z., Wang Z., Zhao Z., Lou X., Xia F., Tang B.Z. (2020). Efficient near-infrared photosensitizer with aggregation-induced emission for imaging-guided photodynamic therapy in multiple xenograft tumor models. ACS Nano..

[46] Wang D., Su H., Kwok R.T.K., Shan G., Leung A.C.S., Lee M.M.S., Sung H.H.Y., Williams I.D., Lam J.W.Y., Tang B.Z. (2017). Facile synthesis of red/NIR AIE luminogens with simple structures, bright emissions, and high photostabilities, and their applications for specific imaging of lipid droplets and image-guided photodynamic therapy. Adv. Funct. Mater..

[47] Li L., Zhang L., Tong X., Li Y., Yang Z., Zhu D., Su Z., Xie Z. (2020). Near-infrared-emitting AIE multinuclear cationic Ir(III) complex-assembled nanoparticles for photodynamic therapy. Dalton Trans..

[48] Li Y., Zhang J., Liu S., Zhang C., Chuah C., Tang Y., Kwok R.T.K., Lam J.W.Y., Ou H., Ding D. (2021). Enlarging the reservoir: High absorption coefficient dyes enable synergetic near infrared-II fluorescence imaging and near infrared-I photothermal therapy. Adv. Funct. Mater..

[49] Zhang L., Li Y., Che W., Zhu D., Li G., Xie Z., Song N., Liu S., Tang B.Z., Liu X. (2019). AIE multinuclear Ir(III) complexes for biocompatible organic nanoparticles with highly enhanced photodynamic performance. Adv. Sci..

[50] Wang Z., Liu B., Sun Q., Feng L., He F., Yang P., Gai S., Quan Z., Lin J. (2021). Upconverted metal-organic framework janus architecture for near-infrared and ultrasound co-enhanced high performance tumor therapy. ACS Nano..

[51] Wang Y., Li Y., Zhang Z., Wang L., Wang D., Tang B.Z. (2021). Triple-jump photodynamic theranostics: MnO_2_ combined upconversion nanoplatforms involving a type-I photosensitizer with aggregation-induced emission characteristics for potent cancer treatment. Adv. Mater..

[52] Liu S., Han J., Chang Y., Wang W., Wang R., Wang Z., Li G., Zhu D., Bryce M.R. (2022). AIE-active iridium(III) complex integrated with upconversion nanoparticles for NIR-irradiated photodynamic therapy. Chem. Commun..

[53] Zhao S., Wu S., Jia Q., Huang L., Lan M., Wang P., Zhang W. (2020). Lysosome-targetable carbon dots for highly efficient photothermal/photodynamic synergistic cancer therapy and photoacoustic/two-photon excited fluorescence imaging. Chem. Eng. J..

[54] Li C., Liu J., Hong Y., Lin R., Liu Z., Chen M., Lam J.W.Y., Ning G.H., Zheng X., Qin A. (2022). Click synthesis enabled sulfur atom strategy for polymerization-enhanced and two-photon photosensitization. Angew. Chem. Int. Ed..

[55] Lu S., Zhou F., Zhang Q., Eda G., Ji W. (2019). Layered hybrid perovskites for highly efficient three-photon absorbers: Theory and experimental observation. Adv. Sci..

[56] Xu W., Lee M.M.S., Nie J.J., Zhang Z., Kwok R.T.K., Lam J.W.Y., Xu F.J., Wang D., Tang B.Z. (2020). Three-pronged attack by homologous far-red/NIR AIEgens to achieve 1+1+1>3 synergistic enhanced photodynamic therapy. Angew. Chem. Int. Ed..

[57] Ma H., Zhao C., Meng H., Li R., Mao L., Hu D., Tian M., Yuan J., Wei Y. (2021). Multifunctional organic fluorescent probe with aggregation-induced emission characteristics: Ultrafast tumor monitoring, two-photon imaging, and image-guide photodynamic therapy. ACS Appl. Mater. Interfaces.

[58] McKenzie L.K., Bryant H.E., Weinstein J.A. (2019). Transition metal complexes as photosensitisers in one- and two-photon photodynamic therapy. Coordi. Chem. Rev..

[59] Porrès L., Mongin O., Katan C., Charlot M., Pons T., Mertz J., Blanchard-Desce M. (2004). Enhanced two-photon absorption with novel octupolar propeller-shaped fluorophores derived from triphenylamine. Org. Lett..

[60] Liu J., Jin C., Yuan B., Liu X., Chen Y., Ji L., Chao H. (2017). Selectively lighting up two-photon photodynamic activity in mitochondria with AIE-active iridium(Ⅲ) complexes. Chem. Commun..

[61] Fang F., Yuan Y., Wan Y., Li J., Song Y., Chen W.C., Zhao D., Chi Y., Li M., Lee C.S. (2022). Near-infrared thermally activated delayed fluorescence nanoparticle: A metal-free photosensitizer for two-photon-activated photodynamic therapy at the cell and small animal levels. Small.

[62] Jiang Y., Zhu W., Xu Z., Zhang Z., Tang S., Fan M., Li Z., Zhang J., Yang C., Law W.-C. (2022). A mitochondrion-targeting two-photon photosensitizer with aggregation-induced emission characteristics for hypoxia-tolerant photodynamic therapy. Chem. Eng. J..

[63] Cai X., Wang K.N., Ma W., Yang Y., Chen G., Fu H., Cui C., Yu Z., Wang X. (2021). Multifunctional AIE iridium (III) photosensitizer nanoparticles for two-photon-activated imaging and mitochondria targeting photodynamic therapy. J. Nanobiotechnol..

[64] Zhang Q., Lu X., Cao H., Wang H., Zhu T., Tian X., Li D., Zhou H., Wu J., Tian Y. (2020). Multiphoton absorption iridium(III)-organotin(IV) dimetal complex with AIE behavior for both sensitive detection of tyrosine and antibacterial activity. ACS Appl. Bio. Mater..

[65] Lu X., Xiong C., Li B., Du W., Li D., Ma W., Tian X., Tian Y., Zhang Q. (2022). Three-photon absorption iridium(Ⅲ) photosensitizers featuring aggregation induced emission. Inorg. Chem. Front..

[66] Vakifahmetoglu-Norberg H., Ouchida A.T., Norberg E. (2017). The role of mitochondria in metabolism and cell death. Biochem. Biophys. Res. Commun..

[67] Li X., Zhao Y., Zhang T., Xing D. (2021). Mitochondria-specific agents for photodynamic cancer therapy: A key determinant to boost the efficacy. Adv. Healthc. Mater..

[68] Wang K., Klionsky D.J. (2011). Mitochondria removal by autophagy. Autophagy.

[69] Jin C., Liu J., Chen Y., Guan R., Ouyang C., Zhu Y., Ji L., Chao H. (2016). Cyclometalated iridium(III) complexes as AIE phosphorescent probes for real-time monitoring of mitophagy in living cells. Sci. Rep..

[70] Alam P., Dash S., Climent C., Kaur G., Choudhury A.R., Casanova D., Alemany P., Chowdhury R., Laskar I.R. (2017). ‘Aggregation induced emission’ active iridium(Ⅲ) complexes with applications in mitochondrial staining. RSC Adv..

[71] Sheet S.K., Sen B., Aguan K., Khatua S. (2018). A cationic organoiridium(Ⅲ) complex-based AIEgen for selective light-up detection of rRNA and nucleolar staining. Dalton Trans..

[72] Gupta G., Das A., Ghate N.B., Kim T., Ryu J.Y., Lee J., Mandal N., Lee C.Y. (2016). Novel BODIPY-based Ru(II) and Ir(III) metalla-rectangles: Cellular localization of compounds and their antiproliferative activities. Chem. Commun..

[73] Kucharzyk K.H., Crawford R.L., Cosens B., Hess T.F. (2009). Development of drinking water standards for perchlorate in the United States. J Environ. Manage..

[74] Capen C.C. (1997). Mechanistic data and risk assessment of selected toxic end points of the thyroid gland. Toxicol. Pathol..

[75] Chao D., Ni S. (2016). Highly selective sensing of ClO_4_^−^ in water with a simple cationic iridium(III) complex and its application in bioimaging. J. Photoch. Photobio. A Chem..

[76] Lu F., Yamamura M., Nabeshima T. (2013). A highly selective and sensitive ratiometric chemodosimeter for Hg_2_^+^ ions based on an iridium(Ⅲ) complex via thioacetal deprotection reaction. Dalton Trans..

[77] Chao D., Zhang Y. (2017). A water–soluble cationic Ir(III) complex for turn–on sensing of ClO_4_^−^ based on aggregation–induced emission. Sensor. Actuat. B Chem..

[78] Chakraborty A., Seth D., Setua P., Sarkar N. (2006). Photoinduced electron transfer in a protein−surfactant complex:  Probing the interaction of SDS with BSA. J. Phys. Chem. B.

[79] Bayraktutan T., Onganer Y. (2017). Spectral-luminescent study of coumarin 35 as fluorescent “light-up” probe for BSA and DNA monitoring. Dyes Pigments.

[80] Kachwal V., Sharma P.K., Sarmah A., Chowdhury S., Laskar I.R. (2020). A multistimuli responsive heteroleptic iridium(Ⅲ) complex: Role of hydrogen bonding in probing solvent, pH and bovine serum albumin (BSA). J. Mater. Chem. C.

[81] Fair R.J., Tor Y. (2014). Antibiotics and bacterial resistance in the 21st century. Perspect. Med. Chem..

[82] Ramírez-Castillo F.Y., Loera-Muro A., Jacques M., Garneau P., Avelar-González F.J., Harel J., Guerrero-Barrera A.L. (2015). Waterborne pathogens: Detection methods and challenges. Pathogens.

[83] Bengtsson-Palme J., Kristiansson E., Larsson D.G.J. (2017). Environmental factors influencing the development and spread of antibiotic resistance. FEMS Microbiol. Rev..

[84] Gupta A., Prasad P., Gupta S., Sasmal P.K. (2020). Simultaneous ultrasensitive detection and elimination of drug-resistant bacteria by cyclometalated iridium(III) complexes. ACS Appl. Mater. Interfaces.

